# Polyolefin Recyclates for Rigid Packaging Applications: The Influence of Input Stream Composition on Recyclate Quality

**DOI:** 10.3390/polym15132776

**Published:** 2023-06-22

**Authors:** Moritz Mager, Michael Berghofer, Joerg Fischer

**Affiliations:** Institute of Polymeric Materials and Testing, Johannes Kepler University Linz, Altenberger Straße 69, 4040 Linz, Austria

**Keywords:** polyolefins, mechanical recycling, circular economy, recyclate quality, substitution potential

## Abstract

In order to shift to a circular plastics economy, high quality recyclates are required to effectively substitute virgin materials. Current approaches to empirically quantify the substitutability for recyclates are mainly limited by the abundance of virgin material grades along with a lack of adequate application-specific property profiles. In contrast, this work aims for a holistic analysis of the substitution potential of polyolefin recyclates intended for rigid packaging applications. This approach is fundamentally based on the classification of virgin polyolefins into different application-specific sub-groups with defined property windows derived from supplier data sheets, which allows for a generalization within one polymer type without neglecting the various available material grades. Moreover, the findings should provide valuable information for improvements of quality-defining process steps along the value chain of mechanical recycling. Therefore, it is of great importance to correlate the input stream composition of the investigated recyclates with the obtained qualities. The investigation of the substitution potential for selected recyclates clearly highlights the necessity of functional recycling for enhanced quality levels, which especially affects the sorting step in the recycling value chain. This work illustrates that a homogeneous waste stream directly correlates with a high substitution potential. Thus, the development of economically viable sorting strategies which take the functionality of plastic waste products into account must be targeted in future research. Furthermore, the development of detailed application-specific property windows in a joint effort with manufacturers should be pursued, as it allows for a meaningful empirical quantification of the substitutability for recyclates obtained from mechanical recycling.

## 1. Introduction

Plastics play a central role in circular economy concepts, as increasing amounts of plastics waste necessitate fundamental changes of the linear plastics economy in a sustainable manner [1,2,3]. By a successful implementation of circularity solutions for plastics, benefits are achieved in the environment, economy, and society [4,5]. Closing the loop for polyolefins (POs) is mainly attempted by mechanical recycling, which showed significant advances in technology over the last decades [6,7]. Nevertheless, material characteristics are negatively affected by service life and reprocessing, which is why successful recycling is limited to few cycles with waste streams of high purity [8]. In fact, all parts of the plastics value chain contribute to the final recyclate quality. Hence, additivation, product design, and end-of-life treatment (i.e., collection, sorting, shredding, washing, reprocessing) need to be evaluated to achieve quality improvements [6].

The quality of recyclates is a widely discussed topic, since a lack of general definitions and industrial standards prevails [9,10]. Tonini et al. [11] addressed this issue in their review, aiming for a harmonization of existing approaches for the definition of recyclate quality. Their main findings show that the substitutability of primary materials by secondary materials is a key quality indicator [12,13]. Recyclates are only able to replace virgin plastics if certain requirements can be met. Primarily, these requirements are defined by the intended end-use application and the respective processing technology. Since virgin plastics are tailored for specific applications and processing technologies, functional recycling should be pursued, which intends to retain material specific properties for an increased substitution potential [11,14]. This implies that highly homogeneous waste streams both in terms of polymer type and material characteristics would significantly enhance recyclate quality.

As it is of great importance to this work, light is shed on the quality-defining aspects of waste treatment to ensure high-level secondary materials obtained from mechanical recycling. More specifically, the influence of collection and sorting of plastic waste on the recyclate quality are discussed in detail. Thus, informal recycling, which is commonly conducted in lower-income countries [15,16], is compared to formal recycling on industrial scale, which prevails in high-income countries [17]. Both lower- and high-income countries are specialized in treating mixed material waste (MMW), especially mixed plastic packaging, for recycling. Due to significantly lower costs of operation in waste collection and sorting, informal recycling offers the potential to generate waste streams of high purity [18]. Furthermore, manual sorting decisions enable a broad range of waste fractions being sorted after, with the possibility of taking the functionality or processing technology of products in the waste stream into account. Gall et al. [19] have already found that informally sourced recyclates are able to compete with commercial grades available on the European market. However, the substitution potential is yet to be evaluated. Nevertheless, the availability of high-quality recyclates would be beneficial to boost domestic production, as a locally sourced and cost-effective feedstock would be provided [16].

In the formal system, waste collection of plastic packaging is a service commonly provided at municipal level by door-to-door collection or by central collection points [17]. With only a few exemptions of highly pure mono-material collection schemes (e.g., polyethyene terephthalate (PET) bottles), MMW is eventually treated in sorting facilities [20,21]. Since it is essential for a sorting plant to operate cost-efficiently, the sorting capability is mostly compromised by economical factors such as upfront investments, the costs of energy and labor, as well as the price of virgin materials [22]. Therefore, sorting facilities for mixed plastic packaging are equipped with a limited number of near infrared (NIR) and/or color sorters, consequently limiting the amount of waste fractions being sorted after.

The aim of this work is to investigate the impact of the waste stream composition on the recyclate quality. In this regard, grades of informally sourced PO recyclates, which are defined by a limited heterogeneity due to manual sorting of post-consumer waste, are compared to formal European benchmark recyclates. The materials are subject to rheological, mechanical, thermal, and compositional characterization in order to determine recyclate characteristics. By acquiring application-specific requirements from data sheets of virgin producers, the recyclate quality of the selected grades in terms of substitution potential for rigid packaging applications can be evaluated.

## 2. Materials and Methods

### 2.1. Materials

Within this study, representative polypropylene (PP) and polyethylene high-density (PE-HD) recyclates were selected. They all share the preceding typical mechanical recycling process consisting of sorting, shredding, washing, and re-processing. However, the supply chain of waste collection and sorting differs significantly, since both formal and informal grades of recycled PP (rPP) and recycled PE-HD (rPE) were included in the research. Therefore, the materials can be grouped in informal PP recyclates (i-rPP), formal PP recyclates (f-rPP), informal PE-HD recyclates (i-rPE), and formal PE-HD recyclates (f-rPE). A summary of the materials is given in Table 1, depicting four grades of each group and their respective colors.

Formal recyclates were sourced from recyclers located in Austria and Germany which process pre-sorted plastics waste derived from the separate waste collection system. The selected benchmark recyclates represent the current state of plastics recycling in Europe, which is achieved by automated sorting processes. However, no further information on the composition of the input stream processed in the formal system was available since all sorting decisions were automated [8]. The informal recyclates were kindly provided by Mr. Green Africa (MGA; Nairobi, Kenya). These grades offer the benefit of detailed insights in the input stream composition, as the waste is handpicked and manually sorted by trained staff at the facilities of MGA. Table 2 provides an overview of the respective composition, from which conclusions on the functionality can be made.

Both polymer types of informal recyclates feature two grades characterized by highly homogeneous input streams. This is exemplified by i-rPP_1, which consists only of thermoformed trays. Moreover, i-rPP_2, i-rPE_1, and i-rPE_2 are defined by a purified feedstock including only injection molded products; specifically, chairs, crates, or beverage bottle caps were collected. The remaining informally sourced recyclates are a mixture of different waste plastics groups. For the i-rPP grades, the feedstock compromised food packaging, home appliances, and personal care bottle tops in a certain ratio. While no distinct way of processing can be attributed to the various product groups, PP food packaging is manufactured mostly by thermoforming and injection molding, but also by blow molding to some extent. Both home appliances and personal care bottle tops are predominantly manufactured by injection molding. All categories of the i-rPE grades (i.e., food packaging, motor oil, home care, and personal care) can be assigned to extrusion blow molding with negligible exemptions. It is also noteworthy that all informal recyclates with the exception of i-rPE_2 (consisting of mixed beverage bottle tops) are sorted by a specific color, which is also displayed in Table 1. Thus, the material heterogeneity is further limited. Among the formal recyclates, f-rPP_2, f-rPP_3, f-rPE_1, and f-rPE_2 were also sorted by color. Therefore, a lower heterogeneity can be attributed to them as well.

### 2.2. Product Property Windows and Substitution Potential

**Figure 1:**
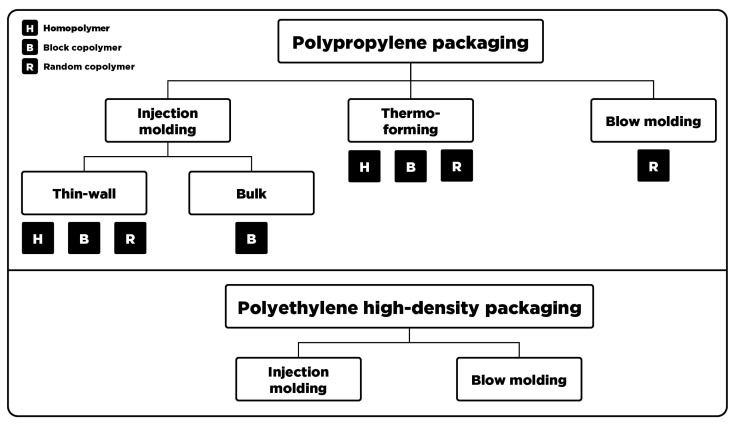
Classifications of virgin polyolefin grades into representative material sub-groups determined by processing method, intended application, and available homo- and copolymers.

In order to determine the substitution potential of recyclates, product property windows of virgin materials had to be defined. A classification into representative virgin material sub-groups was required to obtain meaningful property windows, since the requirements for a wide range of different rigid packaging application result in a versatile product portfolio for both PP and PE-HD. Those sub-groups were determined by the recommended processing method, the intended application, and the available homo- or copolymers in the case of PP. The selection was derived from the product catalog of Borealis (Vienna, Austria) and Lyondellbasell (Rotterdam, the Netherlands) and is depicted in Figure 1. The classification aimed to cover the major processing technologies and applications, which represent the largest share of rigid polyolefin packaging. Thus, specialty polymers, processing methods with low production volumes, and niche applications were left out. Clearly, a higher degree of complexity can be attributed to PP, as a broader product range is covered and both homo- and copolymers are available.

Since the availability of data in technical data sheets (TDSs) varies significantly among different suppliers and also within suppliers’ product portfolios, material properties that were commonly featured in all TDSs had to be selected. Therefore, the product property windows were defined by the melt flow rate (MFR; ISO 1133 [23]), Young’s modulus (ISO 527-2 [24]), and Charpy notched impact strength (nIS; ISO 179-1 [25]). In total, 93 grades of PP and 24 grades of PE-HD were derived from the product catalogs of Borealis (Vienna, Austria) and Lyondellbasell (Rotterdam, the Netherlands) and subsequently categorized according to Figure 1.

**Figure 2:**
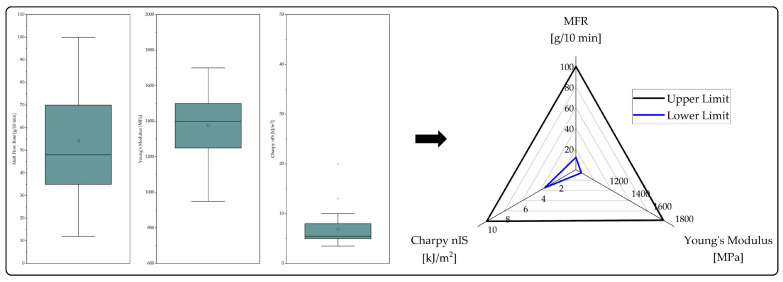
Work flow for the determination of the property windows for each virgin material sub-group: Derivation of upper and lower property limits from box plots.

In Figure 2, the work flow for the determination of the property windows is illustrated. Firstly, the properties of all virgin resins allocated to the respective sub-group were included in a box plot so that outliers, which significantly differ from the remaining data set, could be identified. Secondly, the whiskers, which represent the extremes within the data set excluding the outliers, were selected as upper and lower limits of the product property windows.

By displaying the upper and lower limits in a radar graph, this work aims to draw conclusions on the substitution potential of the selected recyclates, which is indicated by the corresponding recyclate properties. Since MFR, Young’s modulus, and Charpy nIS cover only a limited proportion of relevant material properties, a substantiated conclusion on the substitutability of virgin resins can hardly be determined. However, when considering these three material properties, either a low, moderate, or high substitution potential can be derived from the radar graphs. If the graph shows that the material properties partly or fully lie significantly outside the obtained limits, a low substitution potential is assigned. This means that the recyclate is highly unlikely to replace resins from this virgin material sub-group. A moderate substitution potential is assigned when the limits are not made by at most one material property, indicating that this property can be modified by blending or additivation. If all three material properties of a recyclate initially lie within the limits, a high substitution potential is assigned. It has to be noted that the assignment of a certain substitution potential is strictly based on the limits obtained from TDSs of virgin materials irrespective of the fact that exceeding limits for, e.g., Young’s modulus or Charpy nIS may not diminish the substitution potential of a recyclate under certain circumstances.

### 2.3. Sample Preparation

All materials were provided as pellets. Therefore, no treatment prior to injection molding was required. Multipurpose specimens (MPS) for tensile testing and Type 1 specimens for Charpy impact testing were prepared on an Engel Victory 60 (Engel Austria GmbH, Schwertberg, Austria) according to ISO 19069-2 [26] and ISO 17855-2 [27]. It has to be noted that all rPE grades except i-rPE_1 and i-rPE_2 fall below the MFR limit of 1 g/10 min and specimens thereof should be produced by compression molding in accordance with ISO 17855-2 [27]. Nevertheless, injection molding was selected for all materials as a uniform specimen preparation method with high reproducibility. After molding, the specimens were conditioned at 23 °C and 50% relative humidity for at least 3 days. Notching of the Type 1 specimens was conducted in accordance with ISO 179-1 [25] with a Leica RM2265 microtome (Leica Biosystems Nussloch GmbH, Nussloch, Germany).

### 2.4. Methods

The set of methods utilized in this work includes melt flow rate (MFR) measurements, tensile testing, Charpy impact testing, differential scanning calorimetry (DSC), thermo-gravimetric analysis (TGA), and Fourier-transform infrared (FTIR) spectroscopy in the attenuated total reflection (ATR) mode. The measurement parameters and visualization of test results were chosen in accordance with existing research in this field [28].

MFR was measured with an Mflow melt flow indexer (ZwickRoell, Ulm, Germany) according to ISO 1133-1 [23]. A testing mass of 2.16 kg was selected and measurements were conducted at 230 °C and 190 °C for PP and PE, respectively. Within one measurement that was conducted per material, six cuts were made to obtain meaningful averages and standard deviations.

Young’s modulus was determined in tensile tests using a universal testing machine Zwick/Roell AllroundLine Z020 equipped with Zwick/Roell multi-extensometers (ZwickRoell, Ulm, Germany). According to ISO 527-1 [29] and ISO 527-2 [24], the traverse speed was set to 1 mm/min for the determination of the Young’s modulus and 50 mm/min until breakage. Five specimen per material were tested to obtain meaningful averages and standard deviations.

Charpy notched impact strength was obtained on a Zwick/Roell HIT25P pendulum impact tester equipped with a 2 Joule pendulum according to ISO 179-1 [25]. Ten specimens were tested in edgewise blow direction (ISO 179-1/1eA) for meaningful averages and standard deviations.

Thermal sample analysis was conducted with a DSC 8000 differential scanning calorimeter (PerkinElmer, Waltham, MA, USA). In accordance with ISO 11357-1 [30] and ISO 11357-3 [31], three measurements were conducted for each material with a sample weight of 5 ± 1 mg. The temperature program included a first heating scan from 0 °C to 300 °C, a cooling scan from 300 °C down to 0 °C, and a second heating scan from 0 °C to 300 °C at a heating/cooling rate of 10 K/min. Temperatures significantly above the expected melting temperatures of polyolefins were selected to allow the detection of potential foreign polymers such as polyamides or PET [32]. Nitrogen was used as a purge gas at a constant flow rate of 20 mL/min. The melting enthalpies $\Delta H_{m}$ were determined area of the respective peaks.

Thermo-gravimetric analysis was performed with an STA 6000 simultaneous thermal analyzer (PerkinElmer, Waltham, MA, USA) in a nitrogen atmosphere. Per recyclate grade, two samples with a weight of 25 ± 5 mg each were heated from 30 °C to 900 °C with a heating rate of 20 K/min.

ATR-FTIR spectra were recorded with a spectrum 100 FTIR analyzer (PerkinElmer, Waltham, MA, USA) equipped with an ATR unit with a diamond/ZnSe crystal. After an atmospheric background calibration, surface spectra from three different MPS were recorded for each material. Per measurement, eight scans were conducted in a wavenumber range from 4000 cm^−1^ to 650 cm^−1^ with a spectral resolution of 2 cm^−1^.

## 3. Results

### 3.1. Substitution Potential

**Figure 3:**
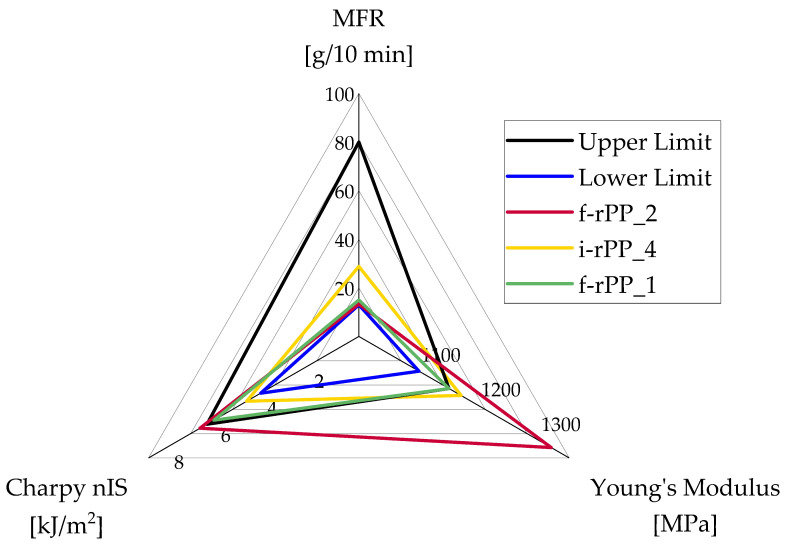
Exemplary radar graph of PP random copolymers designed for thin-wall injection molding packaging. Only f-rPP_2, i-rPP_4, and f-rPP_1 are depicted, as these recyclates represent the three cases of substitution potential in which this work distinguishes.

The determined properties of the investigated recyclates are summarized in Table 3, which are the basis for the evaluation of the substitution potential. An exemplary radar graph of the virgin material sub-group PP random copolymers for thin-wall injection molding packaging is depicted in Figure 3.

As f-rPP_2 significantly exceeds the criteria for Young’s modulus, this grade is considered to have a low potential to substitute virgin resins of this sub-group. While a moderate substitution potential is assigned to i-rPP_4 due to a minor breach of the limits for Young’s modulus, f-rPP_1 fully meets the requirements of this sub-group. Therefore, a high substitution potential is attributed to f-rPP_1.

Since the number of different sub-groups and the simultaneous depiction of all recyclates in the radar graphs hinder a proper data display, a color code is introduced. Hence, a low substitution potential is represented by a red color, a moderate substitution potential is displayed in an orange color, and a high substitution potential is colored green.

#### 3.1.1. Polypropylene Recyclates

**Figure 4:**
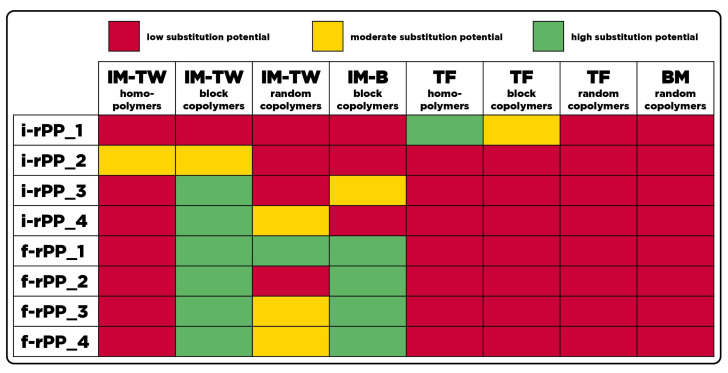
Substitution potential of investigated PP recyclates.

An overview of the substitution potential of the investigated PP recyclates regarding the various virgin material sub-groups is provided in Figure 4. It becomes evident that all recyclates except i-rPP_2 show a high substitution potential in at least one virgin material sub-group. Derived from a homogeneous feedstock of thermoformed trays, i-rPP_1 exhibits a high substitution potential for PP homopolymers for thermoforming applications. Despite an equally homogeneous input stream of injection molded PP chairs, i-rPP_2 does not reach a high substitution potential in any sub-group. A possible explanation would be the presence of fillers or additives, which alter the mechanical properties.

With a high substitution potential in four different sub-groups, f-rPP_1 shows that the property windows of the various sub-groups exhibit overlaps to some extent. Additional requirements for the respective sub-groups must hence be fulfilled to draw conclusions on the actual substitutability of virgin material. However, it is expected that the number of grades with a high substitution potential in certain sub-groups decreases with the incorporation of additional properties. It has to be noted that no conclusion on a superior recyclate quality can be drawn from the fact that a recyclate shows a high substitution potential in multiple sub-groups. Evidently, a high quality recyclate can also be obtained if a high substitution potential is only shown in one particular sub-group, which is the case for i-rPP_1, i-rPP_3, and i-rPP_4.

Interesting findings are obtained by approaching the results from a sub-group level perspective. In theory, a large scale substitution of virgin material can only be achieved if each sub-group features at least one recyclate with a high substitution potential. Undoubtedly, the number of recyclates investigated is limited, but the data clearly show that the requirements for multiple virgin material sub-groups are hardly met by any recyclate. In particular, no recyclate shows a high substitution potential for homopolymers for thin-wall injection molding, as well as block- and random copolymers for thermoforming. This clearly indicates that current recycling systems or more specifically current sorting decisions do not allow for the substitution of raw material in these sub-groups.

Homopolymers in thin-wall injection molding applications are characterized by a Young’s modulus above 1500 MPa, which is an elimination criteria for all recyclates except i-rPP_2. As the majority of the grades included in this study apart from this sub-group do not feature such elevated Young’s moduli, it can be concluded that the low substitution potential throughout the recyclates is caused at least partly by a mixture of different PP grades present in the waste stream. Moreover, the requirements for all three thermoforming sub-groups are only met once by i-rPP_1, which, again, is composed of a pure input stream of thermoformed products. Since no other recyclate shows a high substitution potential in the thermoforming sector, it is assumed that here, the requirements in MFR and mechanical properties are very precise compared to other sub-groups. The opposite can be observed for block copolymers intended for thin-wall injection molding, where only i-rPP_1 exhibits a low substitution potential. In general, a broader property window allows for fewer exclusions of recyclates in terms of potential substitutability within the respective sub-group. Nevertheless, broader product property windows are often a result of manifold possibilities in one product category.

#### 3.1.2. Polyethylene Recyclates

**Figure 5:**
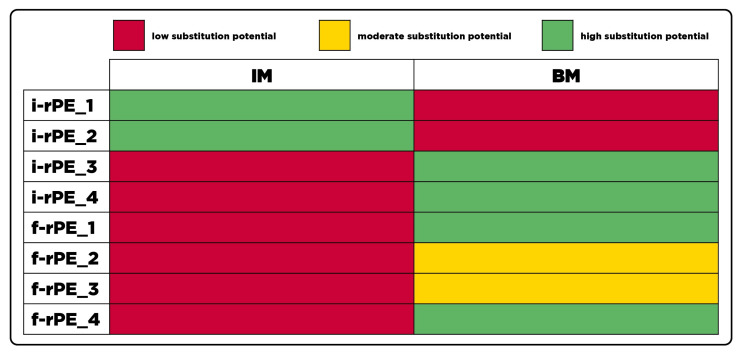
Substitution potential of investigated PE recyclates.

Figure 5 provides an overview on the substitution potential of the PE-HD recyclates covered in this work. The property windows of the two sub-groups significantly differ from each other. While the MFR of injection molding PE-HD may be up to 15 g/10 min (190 °C, 2.16 kg), the range for blow molding grades is limited from 0.25 to 0.4 g/10 min (190 °C, 2.16 kg). Moreover, resins intended for blow molding exhibit superior impact properties compared to injection molding grades. In terms of Young’s moduli, both sub-groups are allocated in the same range of 800–1400 MPa. Since all recyclates were processed into specimens by injection molding irrespective of the MFR, a discrepancy between mechanical data sheet values based on compression molded specimens and the obtained values from injection molded specimens was expected. Mejia et al. [33] observed inferior mechanical properties in injection molded specimens due to a lower degree of crystallinity and defects in the microstructure due to the melt flow in mold filling. Therefore, the lower limit of the Young’s modulus is neglected in the evaluation of the substitution potential if the measured values are insignificantly below 800 MPa, which is the case for i-rPE_3 and i-rPE_4.

Due to the different property windows, none of the recyclates show a high substitution potential in both sub-groups. Both i-rPE_1 and i-rPE_2 are promising to substitute PE-HD resins for injection molding packaging applications. These recyclates are characterized by a homogeneous feedstock based on crates and bottle caps, respectively. The remainder of the investigated recyclates can be allocated to the blow molding sub-group, meaning that no formal PE-HD recyclates for injection molding applications are available. Nevertheless, f-rPE_2 and f-rPE_3 only show a moderate substitution potential as the MFR falls short of the target values for blow-molding applications. This can potentially be caused by cross-linking of polymer chains, which is a potential mechanism in PE-HD degradation [34].

### 3.2. Additional Recyclate Properties

#### 3.2.1. Thermal Properties

**Figure 6:**
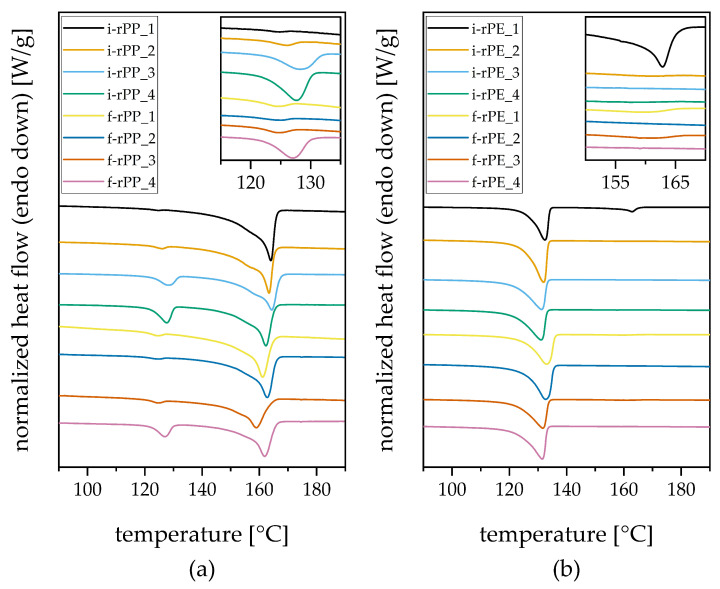
DSC curves with enlarged sections for enhanced visibility of (**a**) PP recyclates and (**b**) PE-HD recyclates, respectively. Stacked curves with an arbitrary shift in vertical direction of the second heating cycle are displayed.

Representative DSC thermograms of the second heating cycle for each investigated material are depicted in Figure 6. Two pronounced melting peaks can be observed for all PP recyclates and for i-rPE_1. From the remaining PE-HD recyclates, i-rPE_4, f-rPE_1, and f-rPE_3 exhibit a barely visible second peak and the rest shows only one peak. In accordance with the literature data, the first melting peak, which is in the range of 124 °C to 133 °C, can be allocated to PE-HD, and the second one, which is between 159 °C and 164 °C, can be identified as a PP melting peak [32]. The corresponding data derived from the DSC measurements are summarized in Table 4, which includes melting peak temperatures and melting enthalpies of the respective peaks.

The thermal properties of polyolefins are significantly affected by their chemical structure and their thermal history [35]. With a second heating step after a controlled cooling step, the thermal history was harmonized for all samples, which allows conclusions on their chemical structure. In this regard, the location and size of the melting peaks must be further evaluated.

In the case of PP, the thermograms greatly differ from homopolymers to copolymers. While homopolymers typically exhibit only one distinct melting peak in the range of 160 °C to 165 °C, the comonomer content is also visible as a second melting peak for block copolymers. Moreover, a shift of the melting peak to significantly lower temperatures is obtained for random copolymers [35]. Therefore, a PE peak does not automatically imply that a PP recyclate is cross-contaminated with PE. This being said, a less pronounced PE melting peak as observed for i-rPP_1, i-rPP_2, f-rPP_1, f-rPP_2, and f-rPP_3 (see Figure 6a) may be caused by the presence of PP block copolymers. Contrary to that, i-rPP_3, i-rPP_4, and f-rPP_4 show larger PE peaks with melting enthalpies in the range of 11 J/g to 15 J/g, which is evidently due to PE cross-contamination. The largest shift of the melting peak was detected for f-rPP_3 with a peak temperature of 158.9 °C. This can be explained by an accordingly larger share of random copolymers present in this material.

The thermal analysis allows evaluation of the crystallinity of the investigated materials, which influences both stiffness and impact behavior on a large scale. However, the calculation of the crystallinity based on the obtained melting enthalpies is only valid for mono-materials [35], as it is strongly influenced by the presence of comonomers, foreign polymers, impurities, or fillers [28]. Therefore, it is not applicable for the investigated recyclates. Nevertheless, the obtained melting enthalpies allow for a qualitative comparisons among the different recyclates, showing that the lowest melting enthalpy is observed for f-rPP_3. This supports the assumption of a high content of random copolymers derived from the melting peak temperature, as random copolymers exhibit lower levels of crystallinity compared to homopolymers and block copolymers.

Besides five PE-HD recyclates showing no PP melting peak, the overall PP cross-contamination seems to be low with PP melting enthalpies of 3.7 J/g and 2.6 J/g for f-rPE_1, and f-rPE_3, respectively (see Table 4). On the contrary, a PP melting enthalpy of 22.4 J/g is detected for i-rPE_1, suggesting that a higher level of PP cross-contamination occurred, potentially due to insufficient sorting [36,37].

#### 3.2.2. Thermo-Gravimetric Properties

**Figure 7:**
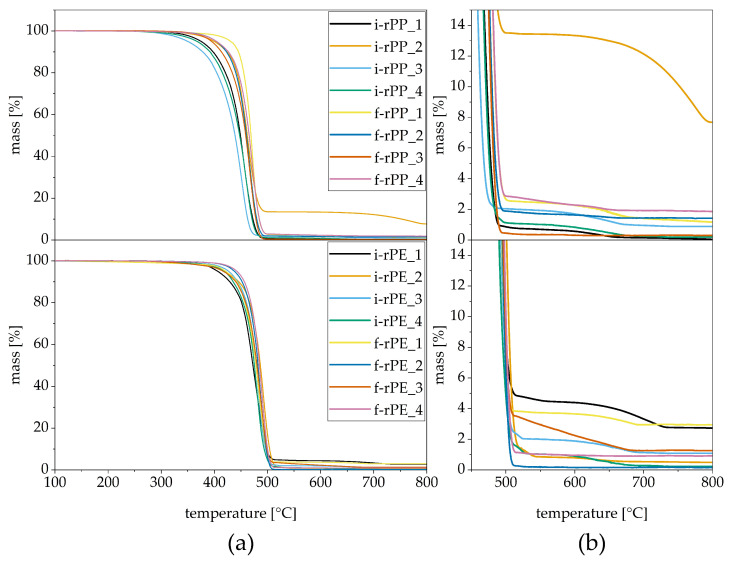
Representative curves obtained in TGA measurements for the investigated recyclates. PP and PE-HD materials are displayed in the upper and lower half, respectively. While the entire temperature range is shown in (**a**,**b**) features a magnified section to enhance the visibility of the pyrolysis residue after the main decomposition step.

Figure 7 shows graphs obtained in TGA measurements. One representative graph is displayed per material (see Figure 7a). A difference in onset temperatures of the polymer backbone decomposition is observed among the PP recyclates, which can be caused by stabilizer residues [38,39]. In general, the inferior thermal stability of PP compared to PE-HD is shown by slightly lower onset temperatures [35].

A magnified section from 450 °C to 800 °C is depicted in Figure 7b to enhance visibility of the residue after the main decomposition step. The pyrolysis residue at 520 °C ranges from 0.4 wt% to 2.9 wt% for PP with the exemption of i-rPP_2, which contained elevated levels of inorganic fillers. Since this recyclate is derived from PP chairs, this finding comes unsurprisingly, as reinforcement materials are needed in order to achieve the required mechanical properties. A step-like mass loss from 13.4 wt% at 520 °C to 7.7 wt% at 800 °C (see Figure 7b, upper half) occurred, which can be attributed to the cleavage of CO_2_ from calcium carbonate (CaCO_3_) [40]. Based on the stoichiometric relation of the molar masses of CaCO_3_ and CO_2_ and the decrease in weight throughout this decomposition step, the initial CaCO_3_ content can be calculated [35]. The calculated amount of 13 wt% of CaCO_3_ is in good agreement with the detected pyrolysis residue after the main decomposition step. While f-rPP_3 exhibits the lowest amount of pyrolysis residue at an almost constant level of 0.4 wt% after the PP backbone decomposition, all other materials show a small-scale decomposition step, which is either linear or pronounced. Gall et al. [28] emphasized in a similar analysis that the exact cause of the decomposition can hardly be determined in heterogeneous recyclates, as a simultaneous decomposition of multiple inorganic residues occurs. Therefore, no quantitative analysis was conducted in this work. However, the pyrolysis residue in recyclates is largely composed of CaCO_3_. Furthermore, the remainder may be composed of talc, carbon black, or glass fiber residue [28].

Similar trends were obtained for the investigated PE-HD recyclates. While the lowest pyrolysis residue was found in f-rPE_2 at 0.2 wt%, i-rPE_1 contained a maximum of 5.0 wt% of foreign material (see Figure 7b, lower half). Based on the second decomposition step which is visible in the TGA curves of all materials except for f-rPP_2, the main inorganic filler present is also most likely CaCO_3_ [40].

#### 3.2.3. Fourier-Transform Infrared Spectroscopy

**Figure 8:**
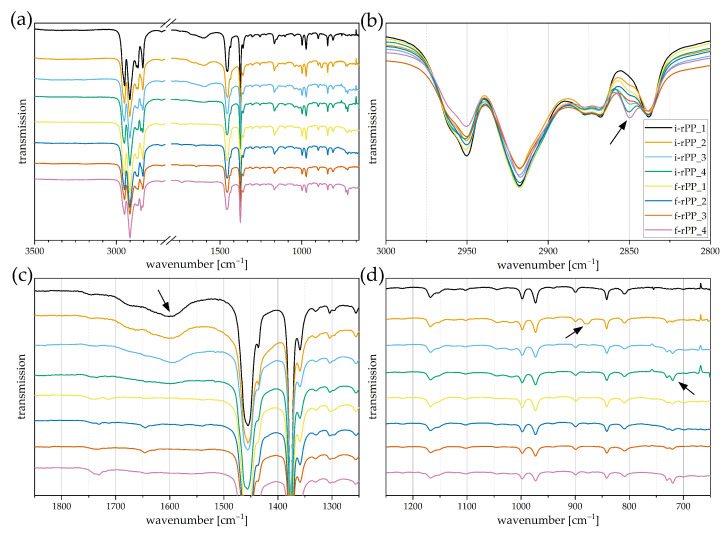
ATR-FTIR spectra of investigated PP recyclates displayed in transmission mode. One representative spectrum per material is shown and the curves are arbitrarily shifted to enhance visibility. (**a**) Full spectra obtained with an interruption between 2700 cm${}^{-1}$ and 1800 cm${}^{-1}$, where no relevant peaks are located, (**b**) magnified region from 3000 cm${}^{-1}$ to 2800 cm${}^{-1}$, (**c**) magnified region from 1850 cm${}^{-1}$ to 1250 cm${}^{-1}$, (**d**) magnified region from 1250 cm${}^{-1}$ to 650 cm${}^{-1}$. Arrows are used to mark absorption bands of interest.

Data generated by Fourier-transform infrared spectroscopy can be used to conduct a detailed compositional analysis of investigated materials [28]. However, the dependency of the waste stream composition and the resultant variety among each batch impede universal conclusions. With regards to this work, the scope of this evaluation was hence set primarily to qualitatively support previous findings obtained in the thermal characterization methods. Therefore, indications of PE/PP-cross contamination and common fillers such as CaCO_3_ are investigated. Furthermore, absorption bands in the carbonyl area are discussed, as they indicate polyolefin degradation. The FTIR spectra of the investigated PP recyclates are displayed in Figure 8 with arbitrary shifts in vertical direction for enhanced visibility. The entire spectra are depicted in Figure 8a with an interruption between 2700 cm^−1^ and 1800 cm^−1^, where no peaks of interest are located. Figure 8b–d provide magnifications of specific regions of interest. Relevant absorption bands are indicated by arrows.

A clear resemblance can be determined between the obtained spectra and those of typical PP virgin materials (Figure 8a) [41,42,43]. In accordance with the results obtained from DSC measurements, the chemical fingerprints of i-rPP_3, i-rPP_4, and f-rPP_4 indicate the presence of PE, as two superimposed peaks at 2850 cm^−1^ and 2838 cm^−1^ are observed (see Figure 8b). These peaks can both be attributed to the symmetric C-H stretching vibration of a CH_2_-group; however, the peak at 2850 cm^−1^ only appears for PE [44,45]. Furthermore, a twin peak at 730/720 cm^−1^ is detected with different intensities, which is also typical for PE (see Figure 8d) [41,44,45]. As mentioned before, the presence of copolymers is accompanied by a small-scale PE content [35], which is why the PE absorption band is observed in all materials. Nevertheless, the respective peaks are most pronounced for i-rPP_3, i-rPP_4, and f-rPP_4, concluding that the PE content must be highest as well, complementing the DSC results.

The results obtained in TGA measurements indicated the presence of CaCO_3_, which was estimated to be as high as 13 wt% in i-rPP_2. The corresponding absorption band of CaCO_3_ is detected at 872 cm^−1^ [46]. As expected, all materials besides i-rPP_2 show minor peaks while i-rPP_2 exhibits a pronounced absorption band (see Figure 8d).

Since polyolefins are prone to thermo-oxidative degradation, the carbonyl band observed in FTIR analysis allows conclusions to be drawn on the level of degradation in both PP and PE [47]. The corresponding absorption bands are found in the range of 1850 cm^−1^ to 1600 cm^−1^ [43,47], whereas larger flat peaks can be observed for i-rPP_1, i-rPP_2, and i-rPP_3 (see Figure 8c). These materials exhibit an advanced degradation compared to the remaining recyclates, for which only comparatively small peaks were detected in this wavenumber range.

**Figure 9:**
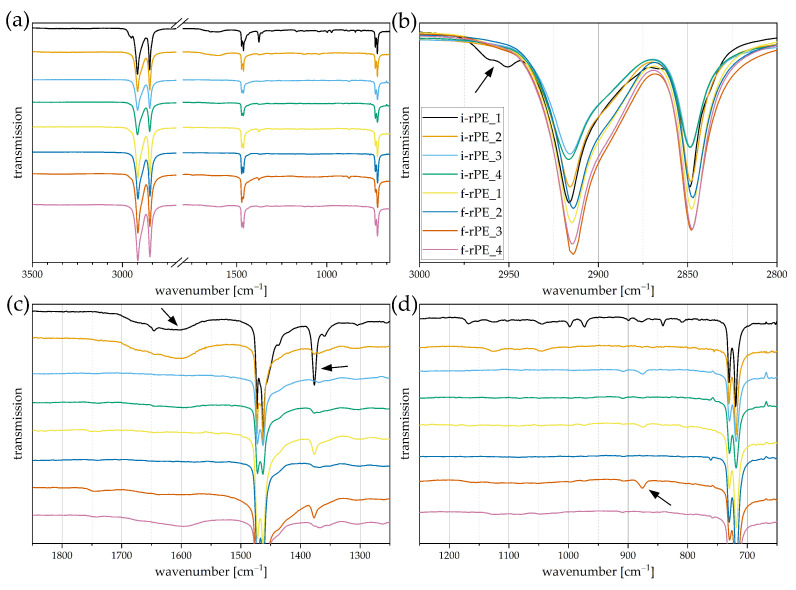
ATR-FTIR spectra of investigated PE recyclates displayed in transmission mode. One representative spectrum per material is shown and the curves are arbitrarily shifted to enhance visibility. (**a**) Full spectra obtained with an interruption between 2700 cm${}^{-1}$ and 1800 cm${}^{-1}$, where no relevant peaks are located, (**b**) magnified region from 3000 cm${}^{-1}$ to 2800 cm${}^{-1}$, (**c**) magnified region from 1850 cm${}^{-1}$ to 1250 cm${}^{-1}$, (**d**) magnified region from 1250 cm${}^{-1}$ to 650 cm${}^{-1}$. Arrows are used to mark absorption bands of interest.

Figure 9 shows the FTIR spectra obtained for the investigated PE-HD recyclates. The style of display is identical to the investigated PP recyclates depicted in Figure 8. Most notably, the high PP content of i-rPE_1 detected in DSC measurements is reflected by a pronounced shoulder between 2975 cm^−1^ and 2930 cm^−1^ (see Figure 9b). This shoulder appears in conjunction with a distinct peak at 1377 cm^−1^ (see Figure 9c) and can be attributed to different vibration modes of the CH_3_ group [42]. The latter is also found in f-rPE_1 and f-rPE_3 with a lower intensity, which is in accordance with the obtained PP melting enthalpies (see Table 4). Furthermore, it could be confirmed that all recyclates except f-rPE_2 contain small levels of CaCO_3_, as absorption bands with different intensities are detected at 872 cm^−1^ [46].

Similar to the results obtained for the PP recyclates, the thermo-oxidative degradation can also be detected by absorption bands between 1850 cm^−1^ and 1600 cm^−1^ [43,47]. While i-rPE_3, f-rPE_1, and f-rPE_1 show almost no sign of degradation, pronounced peaks can be observed for i-rPE_1, i-rPE_2, and f-rPE_4 (see Figure 9c).

## 4. Discussion

In recent years, the great relevance of an adequate description of the substitutability for recycled plastic materials was demonstrated by an increasing number of publications addressing this issue [12,48,49,50]. Primarily, the determination of the substitutability of virgin materials by recyclates plays a key role in life cycle assessments (LCAs), which aim to evaluate potential environmental impacts of plastics derived from different feedstocks [51]. Vadenbo et al. [12] therefore provided a reproducible framework for the substitution modeling of feedstock scenarios to obtain comparative LCAs of waste management systems. Based on their work, multiple empirical models were designed to quantify substitutability, correlating the quality of a recyclate to designated application-specific property profiles [48,49,50]. A fundamental part of these models is the technical substitutability, which represents the functional equivalence between virgin and recyclate materials for a certain application [12,49].

Demets et al. [48] questioned the feasibility of a generalized comparison between virgin plastics and their recyclate counterparts due to the abundance of available material grades within one polymer type. Therefore, their model based on various scoring functions was exemplified in a case study limited to three commercially available PE recyclates, which were regarded as high-quality recyclates for their intended application (i.e., films, bottles, injection molded rigids). The quality of the rPE materials was assessed for four different applications (i.e., films, bottles, injection molded rigids, and injection molded flexibles) and a good substitutability was issued for the intended application, demonstrating the importance of a defined feedstock. In conclusion, Demets et al. [48] highlight the necessity of an extensive property-application matrix that allows for a broader substitutability evaluation.

In contrast to other works, this study does not aim for an empirical quantification of the technical substitutability, but for a generalized approach to assess the technical functionality of polyolefin recyclates intended for rigid packaging applications. Moreover, the findings should provide valuable information for improvements of quality-defining process steps along the value chain of mechanical recycling. Therefore, it is of great importance to correlate the input stream composition of the investigated recyclates with the obtained qualities. This approach is fundamentally based on the classification of virgin polyolefins into different application-specific sub-groups, which allows for a generalization within one polymer type without neglecting the various available material grades (see Figure 1). Similar to studies conducted in the past, processing relevant properties (i.e., MFR) as well as mechanical characteristics (i.e., Young’s modulus and Charpy nIS) serve as indicators for the substitutability. Since these three material properties are regarded as an insufficient representation of a holistic application-specific property profile, the quality evaluation of this work results in the determination of a material’s substitution potential. The substitution potential acts as an indicator for the actual substitutability, specifically enabling the identification of virgin material sub-groups with a lack of high-quality recyclate counterparts.

The investigation of the influence of the waste stream composition on the substitution potential requires specific information on the feedstock of the various recyclates. All formal recyclates were obtained by sensor-based sorting of mixed post-consumer waste, for which no additional information on the exact composition was provided. Therefore, it can be assumed that in the case of the formal PP recyclates all NIR-detectable PP products were sorted with a certain accuracy irrespective of material grades or processing methods. Therefore, an idealized sorting process would result in the market averages of homo- and copolymers as well as additives and fillers to be present in these recyclates. By looking at the properties of the investigated formal recyclates, it becomes apparent that the non-functional sorting results in a similar range of MFR, Young’s modulus, and Charpy nIS at 13.3–17.4 g/10 min, 1090–1320 MPa, and 6.1–6.8 kJ/m^2^, respectively. In regard to the overall property range observed for virgin PP, these intermediate values limit the potential applications significantly, as a greater variation of properties is required to cover the full spectra of PP virgin materials. In terms of substitution potential, all formal PP recyclates exhibit a low potential for homopolymers intended for thin-walled injection molding, all three sub-groups of thermoformed materials, and blow molded packaging. However, the performance of the formal recyclates fulfills the criteria of block copolymers intended for both thin-wall and bulk injection molded packaging, for which a high substitution potential was determined (see Figure 4). Although the requirements are met, a further evaluation of other application-specific properties must be conducted in order to draw conclusions on the actual substitutability.

In contrast to the formally sourced recyclates, the waste stream composition of the informal recyclates was disclosed to a certain extent (see Table 2). These materials can be classified by an either homogeneous feedstock (i.e., i-rPP_1, i-rPP_2) or a heterogeneous composition with information on the relative share of certain product groups (i.e., i-rPP_3, i-rPP_4). The findings obtained for r-rPP_1 resemble those of Demets et al. [48], as this recyclate was derived exclusively from thermoformed trays and exhibits a high substitution potential for homopolymers intended for thermoforming applications. Nevertheless, the equally homogeneous PP recyclate i-rPP_2 did not show a high substitution potential for any virgin material sub-group of packaging. This is most likely caused by the fact that it was derived from a non-packaging application (i.e., chairs), where high levels of CaCO_3_ were used (see Figure 7), ultimately altering the material properties. Moreover, the heterogeneous composition of i-rPP_3 and i-rPP_4 was reflected in a low or moderate substitution potential for all sub-groups except for block copolymers intended for thin-wall injection molding. Since this sub-group is characterized by lower requirements for the investigated properties, the overall recyclate quality is rather poor despite the defined waste stream composition. This clearly shows that a mixture of waste products with different processing origins ultimately results in intermediate material qualities not suitable for the use in specific applications.

The degree of complexity for the evaluation of the investigated PE-HD recyclates was lower compared to rPP, as the virgin PE-HD materials were classified in just two different sub-groups. It becomes evident that both the informal and formal recycling process bear the potential to yield recyclates with a high substitution potential for one of the two sub-groups. However, only a moderate substitution potential was obtained for f-rPE_2 and f-rPE_3 intended for blow molding applications due to a critically low MFR. It has to be emphasized that none of the formal recyclates achieved a high substitution potential for injection molding applications, as their MFR was below the lower limit in all cases. The informal recyclates suitable for injection molding were derived from either crates or bottle caps, which are potential products to be targeted in industrial sorting facilities in order to generate an injection-molding-specific feedstock. However, a quantitative analysis would be required to verify the economical viability of generating such a feedstock. While the input stream composition of the informal PE-HD recyclates is defined by a high homogeneity, the results obtained for the formal PE-HD recyclates indicate an equal level of homogeneity. Therefore, either the sorting after blow molded PE-HD must already be sufficiently adjusted or the relative share of products of this kind in the waste stream must be sufficiently high to cover potential property changes induced by PE-HD products of other origins.

**Figure 10:**
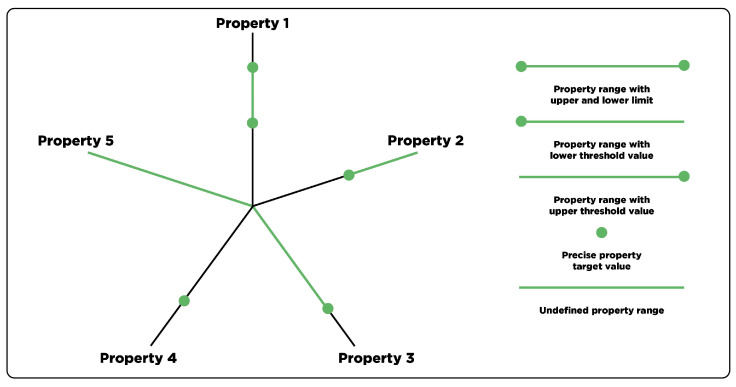
Illustration of an application-specific property profile with different types of potential property windows.

While the substitution potential is indicative of the recyclate quality, a comprehensive evaluation solely based on MFR, Young’s modulus, and Charpy nIS cannot be accomplished. Therefore, the range of quality-defining properties must be further expanded to adequately assess the substitutability for recyclates. The abundance of different material grades and applications implies an equally diverse range of properties that are required for a certain application. Besides the properties investigated in this work, additional material parameters such as color, transparency, odor, hygroscopicity, tensile strength, strain-at-break, or long-term behavior may significantly impact a material’s quality with regard to potential applications [5,52,53]. Moreover, parameters of the extended recyclate characterization presented in this work such as thresholds for the PE/PP cross-contamination or the pyrolysis residue can be incorporated. In this regard, a schematic illustration of a potential application-specific property profile is provided in Figure 10.

While this work defined both the upper and the lower limit for each investigated material property according to supplier data sheets (see property 1 in Figure 10), this simplification may not be applicable for all material characteristics. Huysveld et al. [49] emphasize the redundancy of an upper limit for properties such as Charpy nIS, tensile strength, or strain-at-break, since higher values above a certain threshold bring no disadvantage (see property 2 in Figure 10). The opposite can be observed for property 3 in Figure 10, which exhibits only an upper threshold value. This may be applicable for the Young’s modulus of products that require a certain amount of flexibility [49]. Moreover, a precise property target value can be required (see property 4 in Figure 10), which can be the case for precise color specifications. Finally, property 5 in Figure 10 represents any additional material characteristics without specific target values, which can be included to obtain a holistic material property profile. It must be noted that this illustration is focused on single-point data values as multi-point data such as viscosity measurements would further increase the complexity of defining adequate property windows.

## 5. Conclusions and Outlook

A comprehensive assessment of the substitution potential is key in closing the loop for plastics. With regard to the findings presented in this study, the development of holistic application-specific property windows in a joint effort with manufacturers should be pursued, as it allows for a meaningful empirical quantification of the substitutability for mechanically produced recyclates. While detailed application-specific property profiles are required on a product level, quality enhancing measures derived from the substitutability assessment must be effectively enforced within the recycling value chain. The importance of high-quality recyclates obtained from mechanical recycling is underlined by the framework for a circular plastics economy set by the European Union in recent years, which was recently adapted by a proposal for a mandatory recyclate content in plastic packaging as part of the revision of the packaging and packaging waste directive 94/62/EC [54,55]. With regards to polyolefins, the recyclate content for food-grade products must be at 10% and 50% by 2030 and 2040, respectively, and at 35% and 65% for non-food packaging by 2030 and 2040, respectively. While the suitability of mechanical recycling of polyolefins for food-grade applications has yet to be proven, a supply of high-quality recyclates obtained from mechanical recycling is indispensable for the non-food packaging sector.

The investigation of the substitution potential of selected recyclates for rigid packaging applications clearly highlights the necessity of functional recycling for enhanced quality levels, which affects especially the sorting step in the recycling value chain [11,14]. This work illustrates that a homogeneous waste stream directly correlates with a high substitution potential. Thus, the development of economically viable sorting strategies that take the functionality of plastic waste products into account must be pursued. This work enables the identification of virgin material sub-groups with a lack of high-quality formal recyclate counterparts. Specifically, a low substitution potential was determined for thin-wall PP packaging of high stiffness, PP thermoforming and blow molding products, as well as injection molded PE-HD packaging, which must be targeted in future research. Nevertheless, improvements are required throughout the plastics value chain, starting with the design of packaging products to ensure recyclability. Especially with demanding requirements for high-end applications such as food-contact materials, all waste processing steps must be evaluated and optimized, aiming for best-quality recyclates. In order to accomplish these levels of quality, the recycling process requires comprehensive standardization and certification, which provide the essential framework for a transition to a circular economy. Finally, compounding with additives or blending partners bears the potential to modify recyclate properties to the desired target values, which would enhance substitutability.

## Figures and Tables

**Table 1:** Overview of informal and formal rPP and rPE grades used in this work.

Informal Recyclates	Color	Formal Recyclates	Color
i-rPP_1	natural	f-rPP_1	grey
i-rPP_2	blue	f-rPP_2	white
i-rPP_3	white	f-rPP_3	natural
i-rPP_4	red	f-rPP_4	black
i-rPE_1	blue	f-rPE_1	white
i-rPE_2	grey	f-rPE_2	natural
i-rPE_3	yellow	f-rPE_3	black
i-rPE_4	white	f-rPE_4	grey

**Table 2:** Input stream composition and color of the informal recyclates provided by Mr. Green Africa.

Material	Composition
**i-rPP_1**	100% trays
**i-rPP_2**	100% chairs
**i-rPP_3**	85% food packaging, 10% home appliances, 4% personal care bottle tops, 1% others
**i-rPP_4**	85% home appliances, 10% food packaging, 4% personal care bottle tops, 1% others
**i-rPE_1**	100% crates
**i-rPE_2**	100% beverage bottle caps
**i-rPE_3**	94% food packaging, 2% motor oil, 4% others
**i-rPE_4**	75% home care, 10% food packaging, 10% personal care, 5% motor oil

**Table 3:** Averages of melt flow rate, Young’s modulus, and Charpy notched impact strength of the investigated recyclates.

	MFR	Young’s Modulus	Charpy nIS
	[g/10 min]	[MPa]	[kJ/m${}^{2}$]
**i-rPP_1**	5.6	1490	4.4
**i-rPP_2**	23.4	1670	3.3
**i-rPP_3**	22.1	1090	8.0
**i-rPP_4**	28.8	1170	4.8
**f-rPP_1**	15.0	1150	6.2
**f-rPP_2**	13.3	1320	6.8
**f-rPP_3**	17.7	1090	6.6
**f-rPP_4**	14.9	1150	6.2
**i-rPE_1**	5.8	1210	3.3
**i-rPE_2**	7.0	948	3.6
**i-rPE_3**	0.3	715	20.6
**i-rPE_4**	0.3	673	22.5
**f-rPE_1**	0.3	899	23.5
**f-rPE_2**	0.2	900	26.8
**f-rPE_3**	0.2	818	20.8
**f-rPE_4**	0.3	833	39.9

**Table 4:** Average values of melting peak temperature T${}_{m}$ and the corresponding melting enthalpy ΔH${}_{m}$ for the PE and PP peak, respectively.

	T${}_{\mathrm{mPE}}$	ΔH${}_{\mathrm{mPE}}$	T${}_{\mathrm{mPP}}$	ΔH${}_{\mathrm{mPP}}$
	[°C]	[J/g]	[°C]	[J/g]
**i-rPP_1**	124.4	0.4	163.8	101.2
**i-rPP_2**	125.9	1.3	163.4	86.2
**i-rPP_3**	127.4	11.0	163.6	65.1
**i-rPP_4**	127.9	15.0	162.8	67.7
**f-rPP_1**	124.1	3.7	161.0	90.3
**f-rPP_2**	124.4	1.2	162.6	74.4
**f-rPP_3**	124.5	2.1	158.9	56.7
**f-rPP_4**	126.8	12.1	161.8	59.8
**i-rPE_1**	132.4	140.2	162.8	22.4
**i-rPE_2**	132.2	197.9	-	-
**i-rPE_3**	131.1	188.4	-	-
**i-rPE_4**	131.1	190.4	-	-
**f-rPE_1**	132.8	177.7	160.4	3.7
**f-rPE_2**	132.5	201.4	-	-
**f-rPE_3**	131.3	173.1	159.3	2.6
**f-rPE_4**	131.5	190.8	-	-

## Data Availability

The data presented in this study are available on request from the corresponding author.

## References

[1] Bucknall D.G. (2020). Plastics as a materials system in a circular economy. Philos. Trans. R. Soc..

[2] Hahladakis J.N., Iacovidou E., Gerassimidou S., Letcher T.M. (2020). Chapter 19—Plastic waste in a circular economy. Plastic Waste and Recycling.

[3] Yuan X., Wang X., Sarkar B., Ok Y.S. (2021). The COVID-19 pandemic necessitates a shift to a plastic circular economy. Nat. Rev. Earth Environ..

[4] Ellen MacArthur Foundation Towards the Circular Economy: Economic and Business Rationale for an Accelerated Transition. https://ellenmacarthurfoundation.org/towards-the-circular-economy-vol-1-an-economic-and-business-rationale-for-an.

[5] Shamsuyeva M., Endres H.J. (2021). Plastics in the context of the circular economy and sustainable plastics recycling: Comprehensive review on research development, standardization and market. Compos. Part C Open Access.

[6] Ragaert K., Delva L., Van Geem K. (2017). Mechanical and chemical recycling of solid plastic waste. Waste Manag..

[7] Plastics Europe Plastics—The Facts 2021. https://plasticseurope.org/knowledge-hub/plastics-the-facts-2021/.

[8] Lange J.-P. (2021). Managing Plastic Waste—Sorting, Recycling, Disposal, and Product Redesign. ACS Sustain. Chem. Eng..

[9] Hahladakis J.N., Iacovidou E. (2018). Closing the loop on plastic packaging materials: What is quality and how does it affect their circularity?. Sci. Total Environ..

[10] Eriksen M.K., Damgaard A., Boldrin A., Astrup T.F. (2018). Quality Assessment and Circularity Potential of Recovery Systems for Household Plastic Waste. J. Ind. Ecol..

[11] Tonini D., Albizatti P.F., Caro D., De Meester S., Garbarino E., Blengini G.A. (2022). Quality of recycling: Urgent and undefined. Waste Manag..

[12] Vadenbo C., Hellweg S., Astrup T.F. (2017). Let’s Be Clear(er) about Substitution: A Reporting Framework to Account for Product Displacement in Life Cycle Assessment. J. Ind. Ecol..

[13] Klotz M., Haupt M., Hellweg S. (2022). Limited utilization options for secondary plastics may restrict their circularity. Waste Manag..

[14] Guinée J.B., van den Bergh J.C.J.M., Boelens J., Fraanje P.J., Huppes G., Kandelaars P.P.A.A.H., Lexmond T.M., Moolenaar S.W., Olsthoorn A.A., Udo de Haes H.A. (1999). Evaluation of risks of metal flows and accumulation in economy and environment. Ecol. Econ..

[15] Velis C. (2017). Waste pickers in Global South: Informal recycling sector in a circular economy era. Waste Manag. Res..

[16] Wilson D.C., Velis C., Cheeseman C. (2006). Role of informal sector recycling in waste management in developing countries. Habitat Int..

[17] Kaza S., Yao Lisa C., Bhada-Tata P., van Woerden F. (2018). What a Waste 2.0: A Global Snapshot of Solid Waste Management to 2050.

[18] Hahladakis J.N., Iacovidou E. (2019). An overview of the challenges and trade-offs in closing the loop of post-consumer plastic waste (PCPW): Focus on recycling. J. Hazard. Mater..

[19] Gall M., Wiener M., Chagas de Oliveira C., Lang R.W., Hansen E.G. (2020). Building a circular plastics economy with informal waste pickers: Recyclate quality, business model, and societal impacts. Resour. Conserv. Recycl..

[20] Cimpan C., Maul A., Jansen M., Pretz T., Wenzel H. (2015). Central sorting and recovery of MSW recyclable materials: A review of technological state-of-the-art, cases, practice and implications for materials recycling. J. Environ. Manag..

[21] Gadaleta G., De Gisi S., Binetti S.M.C., Notarnicola M. (2020). Outlining a comprehensive techno-economic approach to evaluate the performance of an advanced sorting plant for plastic waste recovery. Process Saf. Environ. Prot..

[22] Serranti S., Bonifazi G., Pacheco-Torgal F., Khatib J. (2019). 2—Techniques for separation of plastic wastes. Use of Recycled Plastics in Eco-Efficient Concrete.

[23] (2022). Plastics—Determination of the Melt Mass-Flow Rate (MFR) and Melt Volume-Flow Rate (MVR) of Thermoplastics—Part 1: Standard Method.

[24] (2012). Plastics—Determination of Tensile Properties—Part 2: Test Conditions for Moulding and Extrusion Plastics.

[25] (2010). Plastics—Determination of Charpy Impact Properties—Part 1: Non-Instrumented Impact Test.

[26] (2016). Plastics—Polypropylene (PP) Moulding and Extrusion Materials—Part 2: Preparation of Test Specimens and Determination of Properties. https://www.iso.org/standard/66828.html.

[27] (2016). Plastics—Polyethylene (PE) Moulding and Extrusion materials—Part 2: Preparation of Test Specimens and Determination of Properties.

[28] Gall M., Freudenthaler J.P., Fischer J., Lang R.W. (2021). Characterization of Composition and Structure–Property Relationships of Commercial Post-Consumer Polyethylene and Polypropylene Recyclates. Polymers.

[29] (2019). Plastics—Determination of Tensile Properties—Part 1: General Principles.

[30] (2016). Plastics—Differential scanning calorimetry (DSC)—Part 1: General principles.

[31] (2018). Plastics—Differential Scanning Calorimetry (DSC)—Part 3: Determination of Temperature and Enthalpy of Melting and Crystallization.

[32] Domininghaus H., Elsner P., Eyerer P., Hirth T. (2013). Kunststoffe: Eigenschaften und Anwendungen.

[33] Mejia E.B., Mourad A.H.I., Ba Faqer A.S., Halwish D.F., Al Hefeiti H.O., Al Kashadi S.M., Cherupurakal N., Mozumder M.S. (2019). Impact on HDPE Mechanical Properties and Morphology due to Processing. Proceedings of the 2019 Advances in Science and Engineering Technology International Conferences (ASET), Dubai, United Arab Emirates, 26 March–10 April 2019.

[34] Langwieser J., Schweighuber A., Felgel-Farnholz A., Marschik C., Buchberger W., Fischer J. (2022). Determination of the Influence of Multiple Closed Recycling Loops on the Property Profile of Different Polyolefins. Polymers.

[35] Ehrenstein G.W., Riedel G., Trawiel P. (2012). Thermal Analysis of Plastics: Theorie and Practice.

[36] Eriksen M.K., Astrup T.F. (2019). Characterisation of source-separated, rigid plastic waste and evaluation of recycling initiatives: Effects of product design and source-separation system. Waste Manag..

[37] Gall M., Schweighuber A., Buchberger W., Lang R.W., Eriksen M.K., Astrup T.F. (2020). Plastic Bottle Cap Recycling—Characterization of Recyclate Composition and Opportunities for Design for Circularity. Polymers.

[38] Yang J., Huang Y., Lv Y., Zhao P., Yang Q., Li G., Almond J., Sugumaar P., Wenzel M.N., Hill G. (2013). The intrinsic thermal-oxidative stabilization effect of chemically reduced graphene oxide on polypropylene. J. Mater. Chem. A.

[39] Ambrogi V., Cerruti P., Carfagna C., Malinconico M., Marturano V., Perrotti M., Persico P. (2011). Natural antioxidants for polypropylene stabilization. Polym. Degrad. Stab..

[40] Halikia I., Zoumpoulakis L., Christodoulou E., Prattis D. (2001). Kinetic study of the thermal decomposition of calcium carbonate by isothermal methods of analysis. Eur. J. Miner. Process. Environ. Prot..

[41] Noda I., Dowrey A.E., Haynes J.L., Marcott C., Mark J.E. (2007). Group Frequency Assignments for Major Infrared Bands Observed in Common Synthetic Polymers. Physical Properties of Polymers Handbook.

[42] Andreassen E., Karger-Kocsis J. (1999). Infrared and Raman spectroscopy of polypropylene. Polypropylene: An A-Z Reference.

[43] Verleye G.A., Roeges N.P., De Moor M.O. (2001). Easy Identification of Plastics and Rubbers.

[44] Alamo R.G., Mandelkern L., Mark J.E. (2009). Polyethylene, Linear High Density. Polymer Data Handbook.

[45] Gulmine J.V., Janissek P.R., Heise H.M., Akcelrud L. (2002). Polyethylene characterization by FTIR. Polym. Test..

[46] Hummel D.O. (2002). Atlas of Plastics Additives.

[47] Almond J., Sugumaar P., Wenzel M.N., Hill G., Wallis C. (2020). Determination of the carbonyl index of polyethylene and polypropylene using specified area under band methodology with ATR-FTIR spectroscopy. e-Polymers.

[48] Demets R., Van Kets K., Huysveld S., Dewulf J., De Meester S., Ragaert K. (2021). Addressing the complex challenge of understanding and quantifying substitutability for recycled plastics. Resour. Conserv. Recycl..

[49] Huysfeld S., Ragaert K., Demets R., Nhu T.T., Civancik-Uslu D., Kusenberg M., Van Geem K.M., De Meester S., Dewulf J. (2022). Technical and market substitutability of recycled materials: Calculating the environmental benefits of mechanical and chemical recycling of plastic packaging waste. Waste Manag..

[50] Golkaram M., Mehta R., Taveau M., Schwarz A., Gankema H., Urbanus J.H., De Simon L., Cakir-Benthem S., van Harmelen T. (2022). Quality model for recycled plastics (QMRP): An indicator for holistic and consistent quality assessment of recycled plastics using product functionality and material properties. J. Clean. Prod..

[51] Nessi S., Sinkko T., Bulgheroni C., Garcia-Gutierrez P., Giuntoli J., Konti A., Sanye Mengual E., Tonini D., Pant R., Marelli L. (2021). Life Cycle Assessment (LCA) of alternative feedstocks for plastics production, EUR 30725 EN.

[52] Demets R., Roosen M., Vandermeersch L., Ragaert K., Walgraeve C., De Meester S. (2020). Development and application of an analytical method to quantify odour removal in plastic waste recycling processes. Resour. Conserv. Recycl..

[53] Garofalo E., Scarfato P., Di Maio L., Protopapa A., Incarnato L. (2021). Zeolites as effective desiccants to solve hygroscopicity issue of post-consumer mixed recycled polyolefins. J. Clean. Prod..

[54] European Parliament, Council of the European Union Consolidated text: European Parliament and Council Directive 94/62/EC of 20 December 1994 on Packaging and Packaging Waste. https://eur-lex.europa.eu/legal-content/EN/TXT/?uri=CELEX%3A01994L0062-20180704.

[55] European Commission Proposal for a Revision of EU Legislation on Packaging and Packaging Waste. https://environment.ec.europa.eu/publications/proposal-packaging-and-packaging-waste_en.

